# Quantification of hexagonal boron nitride impurities in boron nitride nanotubes *via* FTIR spectroscopy†

**DOI:** 10.1039/c8na00251g

**Published:** 2019-03-12

**Authors:** Haley Harrison, Jason T. Lamb, Kyle S. Nowlin, Andrew J. Guenthner, Kamran B. Ghiassi, Ajit D. Kelkar, Jeffrey R. Alston

**Affiliations:** Joint School of Nanoscience and Nanoengineering, University of North Carolina at Greensboro Greensboro NC 27401 USA; ERC, Incorporated, Edwards AFB CA 93524 USA; Air Force Research Laboratory, Aerospace Systems Directorate, Edwards AFB CA 93524 USA; Joint School of Nanoscience and Nanoengineering, North Carolina A&T State University Greensboro NC 27401 USA jralston1@ncat.edu +1-336-285-2861

## Abstract

Preparation of high-quality boron nitride nanotubes (BNNTs) from commercially available stock is critical for eventual industry adoption and to perform comprehensive experimental studies of BNNTs. Separation of hexagonal boron nitride (h-BN) and BNNTs is a significant challenge, and equally so, quantification of h-BN content in mixed samples is a major challenge due to their nearly identical properties. This work introduces a simple method of quantifying h-BN content in BNNTs based on FTIR analysis. Quantification is achieved by “spiking” a BNNT sample with pure nanoscale h-BN as an internal standard. To demonstrate the efficacy of the quantification technique two BNNT enrichment methods, surfactant wrapping and centrifugation, and a novel sonication-assisted isovolumetric filtration are introduced. FTIR spectra of enriched samples show clear trends throughout the processes. We propose and demonstrate that FTIR peak ratios of the transverse and buckling modes of mixed h-BN/BNNT samples can be used to calibrate and quantify h-BN content in any BNNT sample. Hopefully, this method enables as-received BNNTs to be quantifiably enriched from low purity commercial feedstocks, enabling future development and study of BNNTs and related technology.

## Abstract 2

The peak ratio of in-plane *versus* out-of-plane FTIR absorbance is proportional to weight% of hexagonal boron nitride (h-BN) in boron nitride nanotubes (BNNTs) and can be used to quantify the purity of synthesized BNNTs.

## Graphical abstract

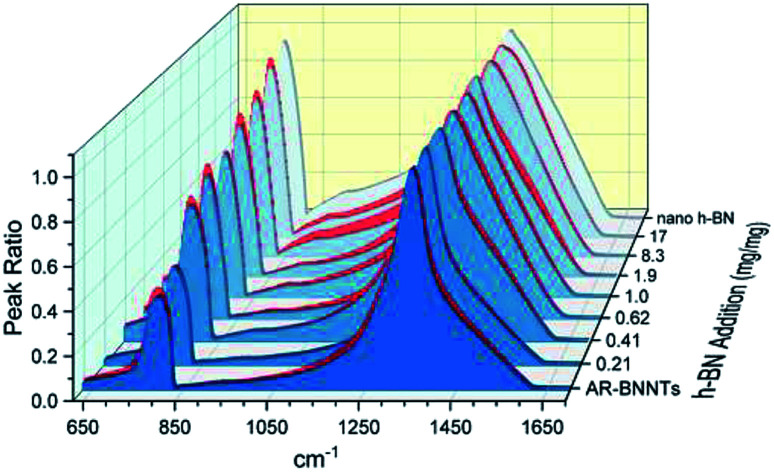

Typical composition of unprocessed commercially available boron nitride nanotubes (BNNTs) is ∼50 wt% BNNTs, ≥20 wt% graphite-like hexagonal boron nitride (h-BN) and amorphous boron and BNHO derivatives.^1,2^ Some BNNT providers offer “purified” materials, however purity claims are weakly supported. High-temperature oxidation followed by water washing have been demonstrated as a suitable method to effectively eliminate amorphous impurities.^3^ However, quantifying and separating h-BN from BNNTs remains a significant challenge. Quantification of h-BN content in mixed h-BN/BNNT samples is a major challenge due to the nearly identical properties of h-BN and BNNTs and often involves laborious use of electron microscopy to assess the sample constitution. In this work, we demonstrate, for the first time, a readily available and practical method of quantifying h-BN sheet content in BNNT samples. The technique is demonstrated after utilizing two different BNNT enrichment methods, surfactant wrapping and centrifugation, and sonication-assisted isovolumetric filtration. Powder XRD analysis and electron microscopy of the enriched samples is shown in the supp. Information and demonstrates the subjective and laborious “current-state” of BNNT purity analysis. We present an internal standard “spiking” technique that utilizes readily available, high purity (99%, <150 nm) nanoscale h-BN and simple FTIR quantification that can be used to calculate the mass percent of h-BN in any BNNT sample. The technique can use any form of FTIR probe for online or offline analysis during BNNT synthesis. This analysis combined with enrichment processes can enable production optimization and ultimately lower cost, readily available commercial BNNT sources.

BNNTs exhibit similar properties to carbon nanotubes (CNTs), but possess much higher chemical and thermal stability, positioning them as important new nanomaterials for future applications in high-temperature composites and components for use in harsh environments.^4–6^ Integrating BNNTs into composite systems designed to withstand challenging environments requires that we study nanotube properties, and ultimately their surface chemistry; so that BNNTs can be made compatible with those composite matrices. To accomplish these studies researchers will need gram-scale quantities of BNNTs of relatively high purity. Much like CNTs, BNNTs can be synthesized as a high purity product, but current synthesis technology is limited to producing a few milligrams of high purity product per batch.^7–11^ The immediate challenge BNNT research faces is the acquisition of sufficient quantities of pure material.

Laboratory-scale synthesis with relatively high BNNT purity is often metal catalyzed,^3,6^ and purification involving acid is needed to remove metal impurities. Removal of amorphous impurities is commonly achieved by oxidation of the sample at elevated temperatures in H_2_O or O_2_ containing atmosphere, which is then removed *via* solvent wash to dissolve B_2_O_3_.^3^ This procedure is effective for laboratory-scale synthesis methods, which produce relatively pure BNNTs where the primary impurities include a low wt% of metal catalyst and small amounts of amorphous BN with a few imperfect BNNTs and h-BN. To produce gram-scale quantities of material, commercial producers are using variations of high temperature and high-pressure vapor synthesis methods with laser or plasma assistance.^10,12–14^ While high quantity production in possible, these processes are not optimized to the extent of producing highly pure material. The most readily available sources, Tekna and BNNT, LLC. offer BNNT feedstock that is less than 50 wt% BNNTs with the remaining composition consisting of hexagonal boron nitride and amorphous BNH derivatives.^1,2^Fig. 1-before is a representative image of as-received commercial grade BNNTs, highlighting the need for further purification. Because metal catalyst is not used to produce these samples, these feedstocks do not require the acid-wash-removal of metal catalyst, however a natural byproduct of this BNNT production processes includes large amounts of h-BN, which have nearly identical chemical and thermal properties to BNNTs.^15^ Amorphous and hexagonal BN is the primary contaminant of commercially produced BNNTs and due to the chemical composition and property similarities to BNNTs, removal h-BN is a major challenge.

**Fig. 1:**
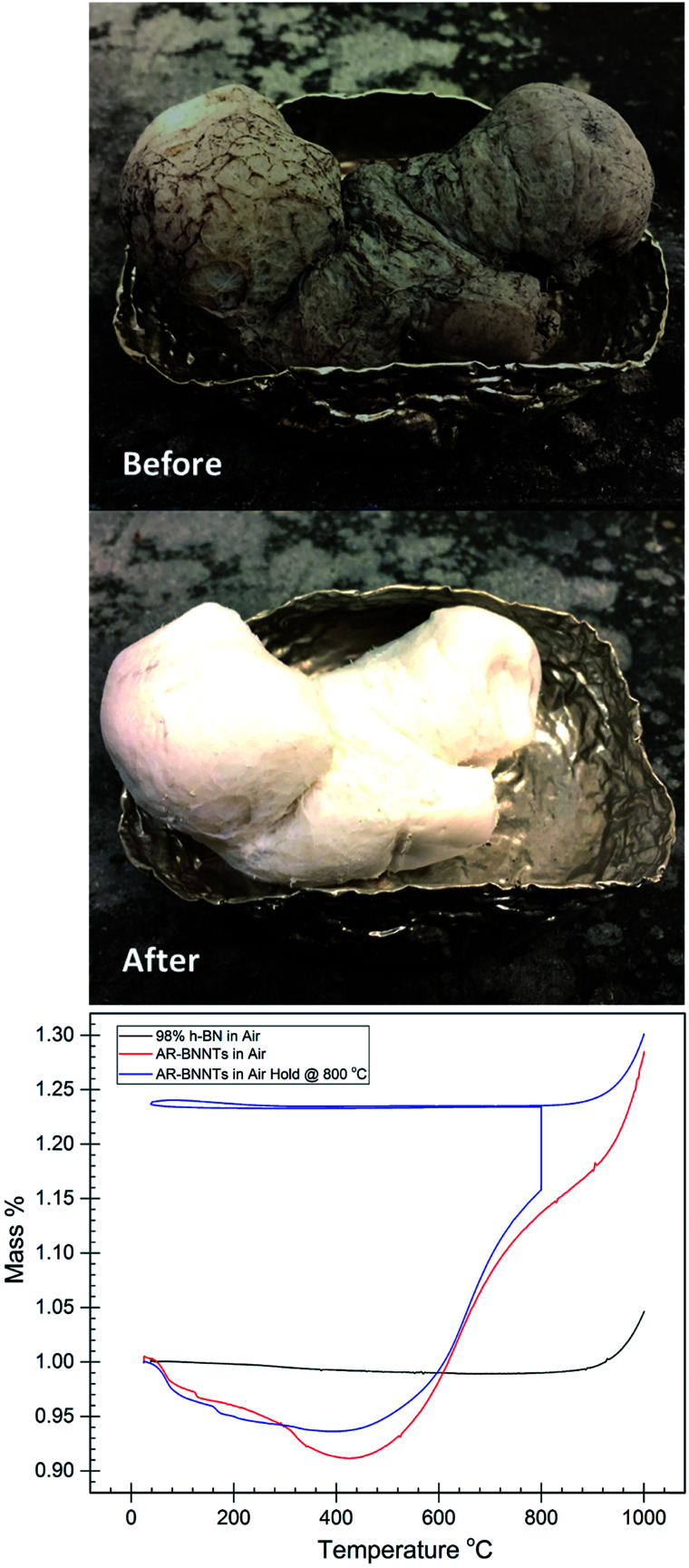
High temperature oxidation of as-received BNNTs and h-BN. Before: as received BNNTs (AR-BNNTs), after: oxidized commercial BNNTs (Ox-BNNTs), AR-BNNTs heated in air at 800 °C for 3 h. Bottom: thermogravimetric analysis of AR-BNNTs (red) and 98% h-BN (black) heated in air at 10 °C min^−1^ up to 1000 °C. AR-BNNTs (blue) heated in air at 10 °C min^−1^ up to 800 °C then held for 3 h, followed by a thermal cycle down to RT and back to 1000 °C at 10 °C min^−1^.

## Removal of amorphous impurities

Large-scale commercial batches of BNNTs can appear grey to light brown in color due to the presence of amorphous boron-containing contaminants and h-BN material (Fig. 1-before). To identify an appropriate oxidation temperature, thermogravimetric analysis (TGA) can be used to observe the mass response of the raw material as it is heated in air (Fig. 1-bottom). During heating to 400 °C, the raw material can lose as much as 10 wt% volatile mass. With further heating in the presence of oxygen amorphous boron oxidizes to B_2_O_3_, and if moisture is present B_2_O_3_ can convert to boric acid. Either process leads to conversion of amorphous material and mass gains approaching 130 wt% of the starting sample. As mass gain plateaus, the amorphous material is becoming fully oxidized, until the temperature climbs towards 800 °C, at which point h-BN and BNNTs begin to oxidize. A sample in which amorphous material is fully oxidized displays a characteristic TGA curve analogous to high purity (98%) h-BN (Fig. 1-bottom). A pure or fully oxidized sample shows a characteristic steady mass up to the onset of h-BN and BNNT oxidation. Based on TGA analysis, a tube furnace set at 800 °C was used to oxidize large quantities of as-received BNNTs (AR-BNNTs). A striking visual transformation occurs (Fig. 1-after) during the oxidation process, changing the AR-BNNTs from grey to a bright white material primarily containing oxidized boron species, h-BN and BNNTs (Ox-BNNTs). At the start of the oxidation process, the sample is plunged directly into the center of the 800 °C tube furnace with an air atmosphere. Rapid oxidation produces the characteristic, however short-lived, flash of boron green flame upon insertion indicating the presence of elemental boron. After oxidation, the bulk material can be washed with warm water or alcohol using gentle bath sonication followed by filtration to effectively dissolve and remove the oxidized material which leaves a sample containing h-BN and BNNTs (OW-BNNTs, where OW is oxidized-washed). ESI Fig. 1† presents full FTIR spectra (4000 cm^−1^ to 500 cm^−1^) of BNNTs at different stages of the oxidation process, highlighting the appearance and removal of oxide in the samples.

## BNNT enrichment

This study describes two methods for the removal of h-BN for the enrichment of OW-BNNT samples: sonication-assisted isovolumetric filtration (SAIF), and surfactant wrapping and centrifugation followed by high-temperature oxidation. SAIF involves the dispersion of OW-BNNTs in a mixture of dimethylformamide (DMF) and acetone. DMF–acetone mixtures have been previously demonstrated as having favorable solvent parameters to promote BNNT disaggregation and dispersion.^16^ A tip-probe ultrasonicator is used to form the initial dispersion which is then transferred to the funnel of a vacuum filtration apparatus. Signal from dynamic light scattering (DLS) of dispersion filtrate through various pore size membranes was used to select a nanopore membrane that selectively passed particles and particle agglomerates with an intensity size distribution near 100 nm. For SAIF to be effective, sonic energy is constantly supplied to the OW-BNNT dispersion while the permeate passes through the membrane. During sonication, the dispersion volume is simultaneously replenished with neat solvent from an addition funnel above the filtration apparatus. A detailed diagram of the SAIF apparatus is provided in ESI Fig. 2.† Shear forces help prevent BNNT and h-BN agglomeration and reaggregation, while the agitation and intense convection produced by the sonication prevents caking on the filter membrane. The small pore size of the membrane (∼200 nm) in concert with the aforementioned dispersion factors creates an environment in which the smaller and lower aspect ratio h-BN particles diffuse through the membrane at a high rate while the high aspect ratio BNNTs impinge on the membrane and redisperse. Removal of h-BN in this way produces a BNNT rich sample (SAIF-BNNTs) that can be collected directly on the membrane. SAIF decreases relative h-BN content as a function of time as smaller particles, h-BN and chopped BNNTs, diffuse through the membrane. However, the risk of damaging BNNTs also increases with time and is exacerbated in the presence of alcohols and water.^17^

Surfactant wrapping and centrifugation, or “density gradient separation”, is a more gentle and effective purification technique previously demonstrated on CNTs.^18^ However, for CNTs, the usefulness of the recovered product can be limited due to the difficulty of completely removing surfactant from the nanotubes.^18–21^ High thermal and oxidative stability offers BNNTs a unique advantage over CNTs for this method and an easy solution to the surfactant removal problem. If metal free surfactants are selected, after centrifugation and sample collection, one can simply heat (400–600 °C) the surfactant-wrapped BNNTs in air to oxidize and volatilize the organic material without damaging the BNNTs. During separation, when centrifugal force is applied to the OW-BNNT dispersion, differences in surfactant interaction and coverage cause differences in buoyancy and will cause BNNTs and h-BN to precipitate from dispersion at different rates.^21^ Supernatant fractions can then be collected and heated to remove the surfactant. Triton X-100 (TX-BNNTs) proved to be the most effective surfactant in this study, producing slightly higher BNNT enrichment than dispersions made with Span 20 and Tween 20.

## Quantification of h-BN content in BNNTs

### X-ray diffraction of boron nitride

**Fig. 2:**
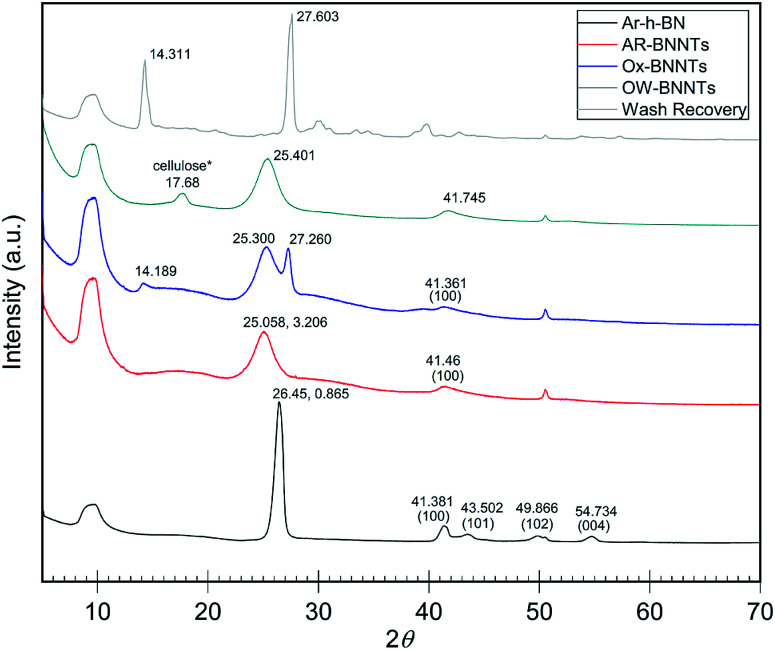
XRD diffractograms of as received commercial h-BN and commercial BNNTs with oxidation and wash products. h-BN peaks near 41°, 43°, 50° and 55° show characteristic 3D ordering.^22^ Broadening and shifting the (002) peak near 26° is a sign of increasing graphitic plane spacing and curvature.^24^ B_2_O_3_ is the primary product removed from the Ox-BNNTs as wash recovery. Note *some cellulose is present due to contamination from sample collection on a cellulose filter membrane. Individual intensity axes are not to scale.

When analyzing BN nanomaterials, distinguishing between BNNTs and h-BN sheets is a major challenge. Analogous studies with graphene and CNTs, carbon analogs of h-BN and BNNTs, respectively, have been conducted exploring this albeit simpler challenge for carbon nanomaterials. Studies employing X-ray diffraction (XRD) show that increased curvature of the graphitic plane (graphene → CNT) leads to decreases in the (002) Bragg plane intensity and results in broadening of the full width at half maximum (FWHM) of the peak. This trend is attributed to the graphitic plane interlayer spacing and manifests similarly in BN analogs.^22^Fig. 2 shows XRD diffractograms of high purity (>98 wt%) h-BN, AR-BNNTs, Ox-BNNTs, and OW-BNNTs. The h-BN diffractogram shows the characteristic set of higher angle peaks due to the three-dimensional order h-BN.^23–25^ AR-BNNTs show the expected broadening for the (002) reflection, indicating a much higher BNNT content (graphitic plane curvature) in the AR-BNNT *versus* h-BN samples.^22^ The (002) 2*θ* for h-BN is 26.45°, compared to 25.06° for as-received BNNTs; and the FWHM is 0.865° and 3.206°, respectively. XRD diffractograms obtained for TX-BNNTs and SAIF-BNNTs are included in ESI Fig. 4.† All show increasing down-shift of the (002) plane and increased broadening, while the Ox-BNNT spectra clearly show the presences of B_2_O_3_.^26^ Crystallization of molten α-B_2_O_3_ at ambient pressure is strongly kinetically unfavored which will limit the amount of diffraction seen in XRD, however characteristic reflections are seen at 14° and 27°. Narrow, higher angle (002) reflections suggest higher content of h-BN, broader and lower angle (002) reflections would suggest increased curvature and increasing three-dimensional disorder from higher content BNNTs.^25^

### Fourier transform infrared (FTIR) spectroscopy of BNNTs and h-BN

FTIR spectroscopy of boron nitride provides us with the crucial insight into the morphology differences of the h-BN and BNNT allotropes, needed to properly quantify h-BN content in BNNT samples. Graphene-like BN nanomaterials, with a hexagonal BN network, present characteristic structural vibrations in the low-frequency region of an FTIR spectra. For stacked sheets of h-BN, two in-plane optical phonon modes, transverse optical (TO) and longitudinal (LO) modes, resonate near 1350 cm^−1^, and are equal in both the *x*- and *y*- axes. Buckling of the hexagonal plane also occurs, and these out-of-plane buckling, (R) modes, resonate near 800 cm^−1^.^9,17,27^ To the best of our knowledge, Chee Huei *et al.*^9^ was first to demonstrate experimentally that when highly pure, monodisperse BNNTs are analyzed, the discrete TO and LO in-plane frequencies can emerge. The LO longitudinal vibrations along the axis resonate sharply at 1369 cm^−1^, and a second signal (1545 cm^−1^) appears for tangential (T) circumferential in-plane modes.^9^ These T modes should be diameter (curvature) dependent but seem to only be visible for highly pure, crystalline BNNTs. More often, for mixed BNNT samples and those with higher h-BN content, T and LO peaks broaden and overlap.^9^ For simplicity, we refer to the broadened, combined peak as TO for this study.

An early computational study by Wirtz *et al.*^28^ offers a detailed explanation of the Raman and IR active modes of h-BN sheets and BNNTs. *Ab initio* calculations from their work predict a diameter dependence of three distinct BNNT radial buckling modes that converge to become the h-BN out-of-plane buckling mode at approximately 818 cm^−1^ as BN tubes becomes more like h-BN sheets, or more simply, the nanotube diameter approaches infinity (*D* → ∞).

The combined computational evidence of Wirtz^28^ and FTIR spectra from pure BNNT synthesis examples^9–11^ inspired us to look more closely at a previously unnoticed spectral feature. It appears that the relative intensity of the out-of-plane *versus* in-plane transmission (R/TO) for pure BNNTs is approximately 95% lower than the equivalent transmissions in pure h-BN. We suspect this is due to the strain induced in the BN bonds as the bending radius of the BN plane increases from h-BN (planar) to smaller and tubes. Straining the bonds of the h-BN sheet would stiffen the out-of-plane buckling modes and reduce translational freedom in the *z*-axis. Translational restriction in the *z*-axis would drastically limit the change in dipole moment that occurs during the buckling stretch, explaining the much lower activity of the absorption and the large difference in R/TO ratio observed for pure BNNTs.

### Measuring unknown [h-BN] using FTIR absorbance and internal standard (spiking)

**Fig. 3:**
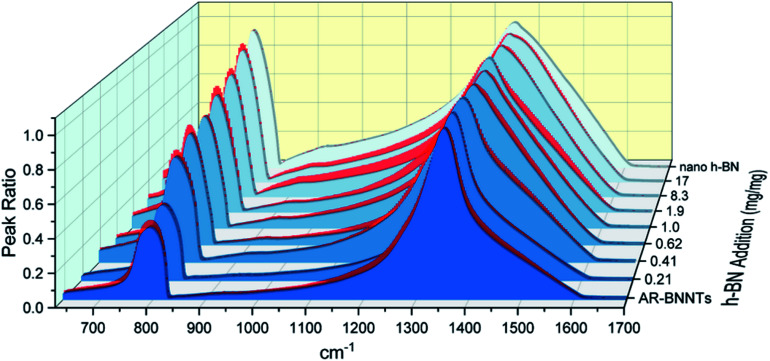
FTIR spectra array of AR-BNNT samples that have been “spiked” with increasing amounts of nanoscale h-BN. Samples are presented from the lowest h-BN concentration (AR-BNNTs) to the highest in the back (pure nanoscale h-BN). The spectra are all normalized to their TO peaks (1350 cm^−1^) to assist visualization of the increasing out-of-plane *versus* in-plane transmission (R/TO) peak ratio as the concentration of h-BN in the sample increases.

**Fig. 4:**
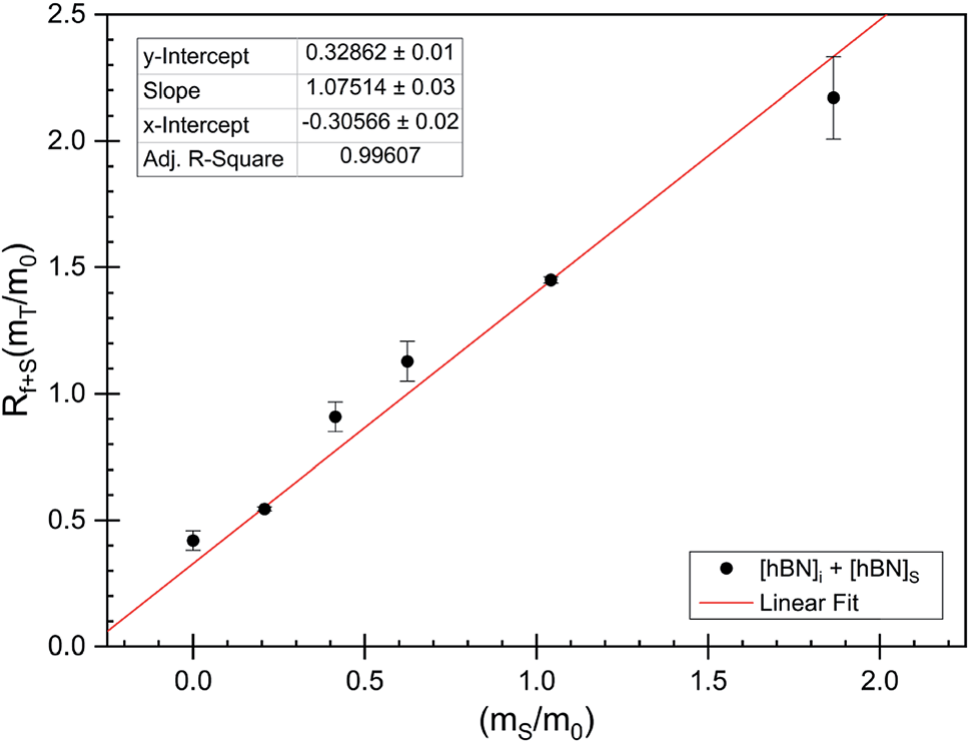
FTIR peak ratios (out-of-plane/in-plane (R/TO) h-BN stretches) of “spiked” samples are plotted *versus* the ratio of h-BN standard added to the as-received BNNTs per eqn (5). The data is fit using non-linear least squares regression (red line) and the *x*-intercept is used to determine the h-BN concentration in the initial as-received BNNT sample per eqn (6).

**Fig. 5:**
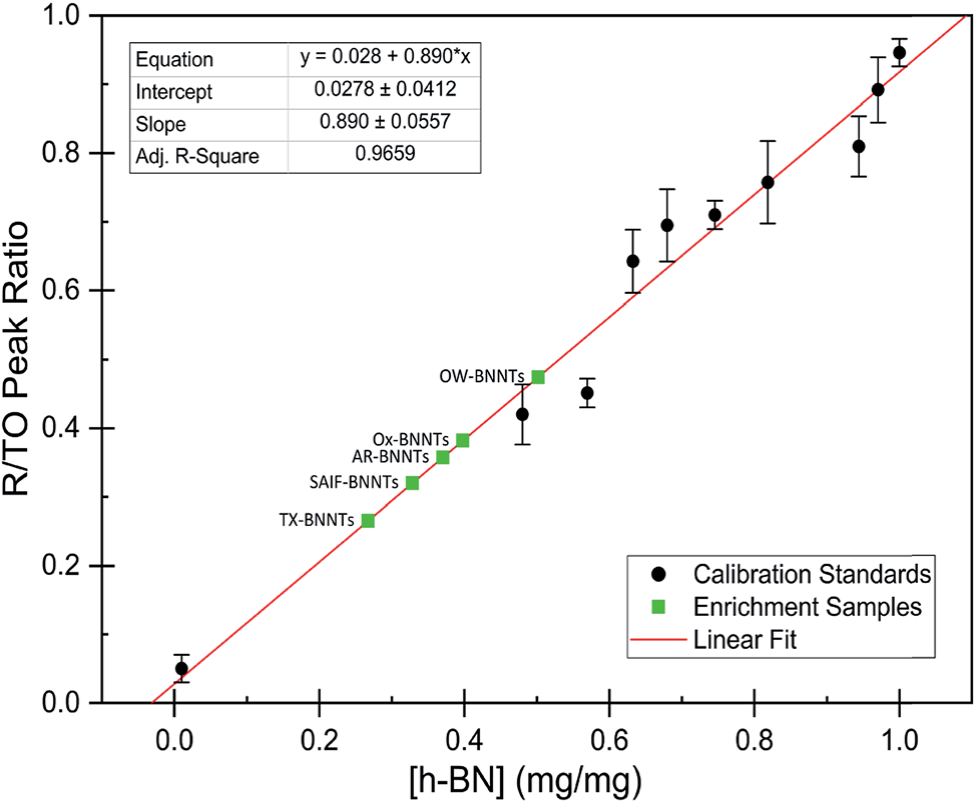
Calibration curve generated from “spiked” standards after determination of [hBN]_i_ in AR-BNNTs. Black circles represent calibration standards. Green squares represent enrichment samples and the calculated weight percent from R/TO peak ratios and based on linear best fit (red line) to calibration curve.

The dramatic difference in low-frequency absorption of pure h-BN *versus* pure BNNTs provides a convenient indicator to be used as a marker for quantifying h-BN content in mixed h-BN/BNNT samples. The linear relationship of the out-of-plane (R) mode *versus* [h-BN] is demonstrated by “spiking” AR-BNNTs with known amounts of pure nanoscale h-BN platelets. By normalizing the FTIR absorbance to the TO peak the effect of increasing h-BN content in BNNT samples can easily be observed. Fig. 3 is a 3-dimensional array of FTIR spectra ranging from unspiked AR-BNNTs to pure h-BN, illustrating the relationship of R/TO peak ratio and h-BN. From this “spiking” technique we also gain the ability to calculate the unknown true concentration of h-BN in a BNNT sample. From the concentration/peak ratio relationship detailed below in eqn (1)–(6), we can use readily available nanoscale h-BN as an internal standard to quantify the amount of h-BN in as-produced BNNTs. Using eqn (5), plotting (*R*_f+S_)(*m*_T_/*m*_0_) *versus* (*m*_S_/*m*_0_), the peak ratio *versus* standard addition is compared (Fig. 4) showing the linear relationship of peak ratio to h-BN content in BNNT samples. From Fig. 4 and eqn (6) we determined the concentration of h-BN in our as-received material was 0.31 ± 0.02 mg h-BN per mg AR-BNNTs. The calculated value of h-BN concentration can then be used to form a calibration curve that can be used to calculate the h-BN concentration of any BNNT sample. Fig. 5 and Table 1 show the calibration curve generated from the “spiked” standards after the initial h-BN concentration was calculated. From the calibration curve it is clear that FTIR R/TO peak ratios can be an effective and straight forward method of quantifying the h-BN content of processed samples. From Fig. 5, we see that as the BNNT samples are enriched from the as-received state (AR-BNNTs) towards BNNT samples with less h-BN content (SAIF-BNNTs and TX-BNNTs), the R/TO ratio decreases towards the spectra of pristine BNNTs, with [hBN] of 0.33 and 0.27 mg h-BN per mg of enriched BNNT sample, respectively (Fig. 5).

**Table 1:** FTIR peak ratios with calculated h-BN weight percent from Fig. 5

Sample	R/TO ratio	h-BN wt%	*σ* ±%
AR-BNNTs	0.357	0.37	0.0248
Chee *et al.*	0.0507	0.0257	0.00351
h-BN	0.98	1	—
Ox-BNNTs	0.382	0.398	0.0265
OW-BNNTs	0.475	0.502	0.0329
SAIF-BNNTs	0.32	0.328	0.0222
TX-BNNTs	0.266	0.267	0.0184
SP20-BNNTs	0.395	0.413	0.0274
TW20-BNNTs	0.52	0.553	0.036

Of further note, is the observation that the frequency of the out-of-plane mode increases (760 cm^−1^ to 814 cm^−1^) as h-BN content is reduced (Fig. 3). The FTIR observations presented herein are in agreement with BN nanotube lattice dynamics calculations where the low-frequency absorption of the spectrum is due to in-plane and out-of-plane modal coupling, and as the sheet rolls into a tube and this characteristic behavior leads to stiffening in the low-frequency tube mode and reduction of the change in dipole moment during stretching.^28^ Our results and observations support the assumption that rolling the h-BN plain into BNNTs stiffens the R mode frequency and can explain the reduced intensity of the low frequency absorption of BNNTs *versus* that of h-BN sheets.

As expected, the measured R/TO ratios are proportional to h-BN weight percent of the measured samples. Table 1 displays the measured R/TO ratios for all the enrichment samples from this study and includes the calculated h-BN weight percent. Fig. 5 is a plot of the enriched samples overlayed on the [hBN] calibration curve with a slope of 0.890 and intercept of 0.028. According to the manufacturer specification, AR-BNNTs are approximately 50 wt% BNNTs and 30 wt% h-BN, with other amorphous material composing the remaining mass.^1^ The measured R/TO ratio for AR-BNNTs is 0.36, which by the equation of the calibration curve is 37 wt% h-BN content. A slightly higher weight percent (50 wt% h-BN) for OW-BNNTs is calculated which is expected and correlates with the removal of amorphous boron during the high-temperature oxidation and washing step. The R/TO ratio and calculation is provided for the Ox-BNNT sample; however, it should be noted that the oxide content of the sample produces FTIR absorptions that overlap with the R and TO peaks, making this method unusable for h-BN wt% calculations for samples with high oxide content. The SAIF process and density gradient separation appears to have been effective at removing a small quantity of h-BN producing enriched BNNT samples of 33 wt% and 27 wt% h-BN, respectively.

## Conclusions

BNNTs are relatively new nanomaterials with high thermo-oxidative and chemical stability, piezoelectric properties, and have a high radiation absorption cross-section. These properties, along with near-mechanical equivalence to carbon nanotubes positions BNNTs as an important component in advance nanocomposites. However, high purity BNNTs have not yet been produced at commercially significant quantities. Until such a time, new techniques to prepare and refine lower quality commercially available stock into high-quality BNNTs will be critical for the future development of BNNT technologies and for our ability to conduct comprehensive experimental studies. While ongoing advances continue to develop and improve the synthesis of BNNTs, enrichment methods will continue to be used, and until now a simple and reliable method to quantify enrichment has been lacking.

This work demonstrates a simple method based on “spiking” any unknown BNNT sample with standard of high purity h-BN which is inexpensive and readily available. FTIR is a versatile measure technique with a variety of probe and instrument arrangements which could possibly allow this measurement technique to be used *in situ* during synthesis to optimize process on the fly. We have shown that *via* FTIR absorbance spectroscopy and the measurement of R/TO peak ratios can be a simple method to quantify the weight % of h-BN in mixed samples. While XRD can also be utilized as a complementary analysis to help distinguish between samples with varying degrees of impurities. In the interest of demonstrating the quantification several crude BNNT enrichments have been shown. From these results we see that surfactant wrapping with centrifugation and sonication assisted isovolumetric filtration are potentially useful methods to enrich low quality commercial BNNT feedstocks. The enrichment methods demonstrated herein have not been optimized in any way.

Boron nitride nanotubes are a newly available commercially produced nanomaterial that offers some exciting qualities and properties that will enable major advancements in composite technology. However, commercial production is a nascent synthesis technology, which has not yet simultaneously produced both large quantities and high purity BNNTs. At the time of publication, several companies have begun offering higher purity BNNTs, enriched through proprietary processes before shipment. However even in these cases quantification of h-BN is subjective and is accomplished *via* tedious combinations of TGA, SEM and TEM analysis. It is our hope with the reporting of this simple FTIR based quantification technique we will enable both commercial and small research labs to be able to optimize their synthesis and enrichment methods, to enable more in-depth studies of BNNTs and more rapid development of BNNT based technologies.

## Methods

### Materials

All materials were purchased and used without further purification. Methanol (99%) and dimethyl formamide (DMF) was purchased from Fisher Scientific. AR-BNNTs were supplied by BNNT, LLC and BNNano. Boron nitride nano platelets (h-BN, 99%) <150 nm particle size was purchased from Sigma Aldrich.

### Oxidation of as-received nanomaterials

88 mg of as-received boron nitride nanotubes (AR-BNNTs) were thermally treated at 800 °C in a Lindberg Blue M tube furnace for 3 hours in air atmosphere. AR-BNNTs are initially gray in color, and after thermal treatment, the fully-oxidized material (Ox-BNNTs) are bright white (Fig. 1), gaining 29 mg in mass. The Ox-BNNTs are dispersed in 100 mL of MilliQ 18.2 MΩ deionized water (DI-H_2_O) and shaken vigorously by hand to further break up the bundles. The mixture is stirred vigorously overnight to fully dissolve B_2_O_3_, followed by vacuum filtration through a 0.8 μm pore-size cellulose membrane to separate the remaining BNNTs and residual h-BN impurities (OW-BNNTs) from the boron oxide solution. The solids are washed on the filter 3 times with 100 mL aliquots of DI-H_2_O, then collected and dried under vacuum at 100 °C for 24 h.

### High-temperature oxidation discussion

The first step toward purification and enrichment of commercially produced BNNTs is a high-temperature oxidation and removal of amorphous material. Oxidation of amorphous boron at room temperature is a kinetically hindered process, but at sufficiently high temperatures (>600 °C) in oxygen-containing atmosphere, amorphous B_*x*_N_*y*_ will rapidly react with O_2(g)_ to form B_2_O_3(s)_ and release N_2(g)_, depending on the B_*x*_N_*y*_ species. Of particular note, the oxidation temperatures used for this study (750–800 °C) were sufficiently high to ignite the boron within the BNNT mass creating a corona of the characteristic green flame emission spectra of elemental boron. Boron oxide softens at 325 °C and melts near 450 °C.^29^ It has been noted^26,30^ that sample weight gain during high-temperature oxidation of boron materials is often significantly lower than expected from the reaction4BN_*x*_ + 3O_2_ → 2B_2_O_3_ + 2*x*N_2_.

B_2_O_3(s)_ is slowly removed from the oxidized material by slow evaporation of B_2_O_3(l)_ during the oxidation process. This is likely due to a significant vapor pressure of B_2_O_3(l)_ at temperatures approaching 800 °C. After high-temperature oxidation removal is achieved *via* hot water washing, which solubilizes B_2_O_3(s)_, as seen in the recovery products of the hot water wash. B_2_O_3_ is clearly visible in the FTIR spectra of unwashed oxidized BNNT material and can be recovered from the wash water (XRD – Fig. 2, FTIR – Fig. SI1†).

### Sonication assisted isovolumetric filtration (SAIF)

OW-BNNTs are added to 50 : 50 vol% DMF : acetone (∼0.25 mg mL^−1^) and ultrasonicated with a 1/4” tapered tip probe at 30% amplitude for 10 minutes to disperse the BNNTs. Membrane porosity was selected by analysis of dynamic light scattering (DLS) data, comparing intensity size distribution of the BNNT dispersion before and after filtration through membranes of decreasing pore sizes. PTFE membrane filters with porosity below 0.45 μm produce significantly lower intensity size distribution, so membranes with pore sizes below 450 nm are suitable for retaining BNNTs. The probe tip is positioned in the dispersion above the filter membrane to minimize damage to the membrane during sonication. The dispersion was continuously sonicated during filtration in a pulsing sequence, alternating ON for 10 seconds, OFF for 15 seconds. Sonication simultaneously maintains BNNT/h-BN disaggregation and provides a forced convection current within the funnel body to keep the long aspect ratio BNNTs from caking on the membrane surface during filtration. As the dispersion was sonicated on the filter membrane a small pressure differential was produced (Δ*P* ∼100 mPa) using a vacuum pump to slowly pull filtrate through the membrane at a controlled flow rate. A neat mixture of 1 : 1 DMF–acetone was simultaneously added to the supernatant to maintain dispersion volume in the filter funnel. During this process, both BNNTs and the remaining h-BN contaminants are well dispersed in the solvent mixture and continuously recirculate throughout the funnel body due to the convection currents produced by sonication. Mass flow through the membrane is driven by the small pressure differential while h-BN selectivity through the filter membrane is based on the combination of pore size selectivity and the large diffusion coefficient difference between high aspect ratio BNNTs and the relatively small h-BN particles.

### Surfactant wrapping

1 wt% solutions of the surfactants (Span 20®, Tween 20®, and Triton X100®) in deionized water DI-H_2_O were added to ∼100 mg of the oxidized and washed BNNTs (OW-BNNTs) in a 100 mL: 50 mg solution to OW-BNNTs ratio. They were stirred vigorously overnight. The following day, the dispersion was centrifuged at 10 G for 10 minutes. The supernatant and solids were collected separately and then dried in a 105 °C oven under nitrogen overnight. The resultant viscous liquid supernatant and powdered solids were further heat treated in a 400 °C tube furnace under a 20 mL min^−1^ air flow to oxidize and volatilize the surfactant and leave behind BNNTs.

### Thermal analysis

Thermogravimetric analysis (TGA) experiments were carried out on a TA Instruments Q5000 TGA under a 20 mL min^−1^ flow of air using high temperature rated platinum sample pans. Heating rates were at 10 °C min^−1^ unless otherwise noted.

### Powder X-ray diffraction (XRD)

XRD experiments were performed at room temperature using a Bruker D2 PHASER equipped with a Cu-K_α_ (*λ* = 1.5418 Å) and a LYNXEYE compound silicon strip detector. Samples were prepared by grinding the material into a fine powder form and placed on a poly(methyl methacrylate) (PMMA) sample holder. Data acquisition was conducted while rotating the sample at 60 rpm, and the pattern was acquired from 5–70° 2*θ* at 2.0° 2*θ* min^−1^, with an integration step size of 0.02° 2*θ*.

### FTIR “spiked” standard preparation

Fourier Transform infrared (FTIR) spectroscopy analysis was performed on a Thermo Scientific Nicolet iS50 FT-IR equipped with a MIRacle Diamond stage and a diamond crystal window attenuated total reflectance (ATR) accessory. The experiments were performed with 32 scans at a resolution of 4 cm^−1^ ranging from 4000–525 cm^−1^. An ambient background scan was taken before each sample acquisition.

To determine the unknown concentration of h-BN in as-received BNNTs, standard samples were prepared. 125 mg h-BN was dispersed in 50 mL MeOH solution followed by pulsed sonication for 20 minutes at 30 W output power from a probe-tip sonicator to disperse the material homogeneously and form a stock nano h-BN solution (2.5 mg mL^−1^). 100 mg AR-BNNTs were dispersed in 20 mL MeOH solution followed by pulse sonication for 20 minutes at 30 W output power to disperse the stock AR-BNNT solution. 10 mL aliquots of the stock AR-BNNT solution were distributed into separate vials. Aliquots of increasing volume of the h-BN stock dispersion were then added to the AR-BNNT sample vials. The mixed dispersions were tip-probe sonicated again for 20 minutes at 30 W output power to homogenize the dispersions. Afterwards, the samples were capped loosely and left in a warm oven (∼50 °C) to evaporate the methanol and leave behind a dry solid AR-BNNT sample “spiked” with a specific amount pure nanoscale h-BN. FTIR spectra of the dry samples is then collected (Fig. 3).

### Determination of unknown h-BN concentration

To calculate the unknown concentration of h-BN in the original AR-BNNT sample we use the following relationships,1
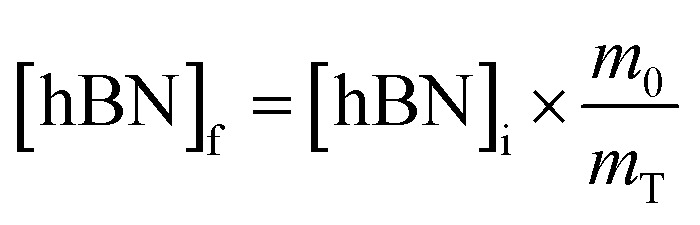
2
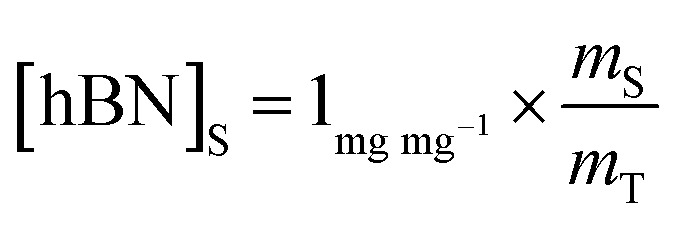
where *m*_0_, *m*_S_, and *m*_T_, are the mass of the AR-BNNTs, mass of “spiked” h-BN, and total mass of the sample in the vial, respectively. [hBN]_i_ is the initial unknown concentration of h-BN in the AR-BNNTs, [hBN]_f_ in the unknown concentration of all h-BN in a “spiked” sample vial after standard addition, and [hBN]_S_ is the known concentration of standard in the “spiked” sample. The initial h-BN concentration [hBN]_i_ in the AR-BNNTs can then be determine by the equivalent ratios;3
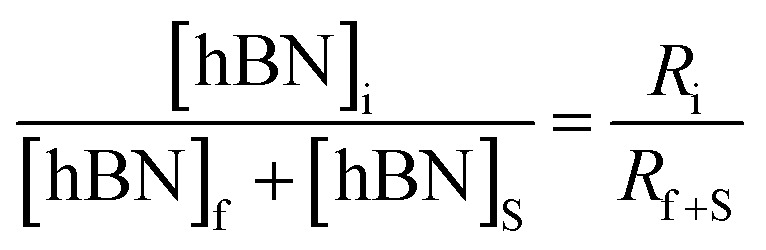
*R*_i_ and R_f+S_ are the initial AR-BNNT R/TO peak ratio and “spiked” ratio, respectively.

Substituting eqn (1) and (2) into (3) gives,4a
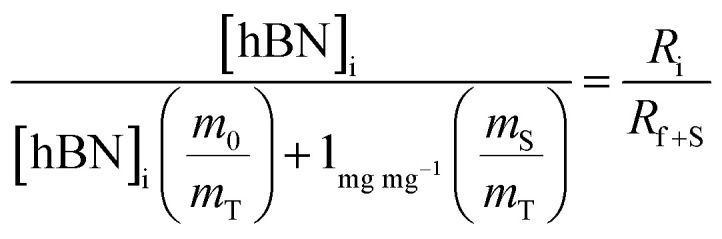
which can be rearranged to4b
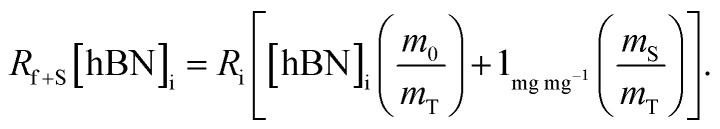
Multiplying both sides by, 
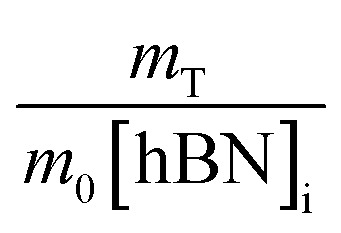
 creates the linear form5
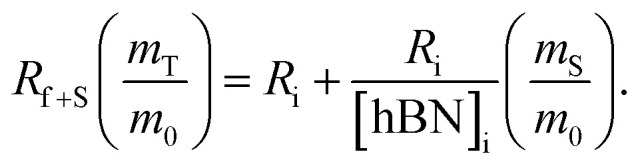


So, by plotting 
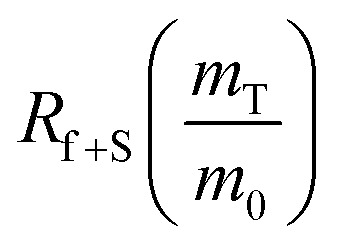
*versus*
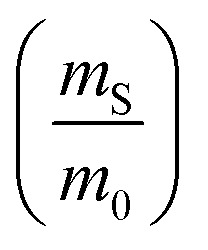
, solving for [hBN]_i_ is found when *y* = 0 by;6a
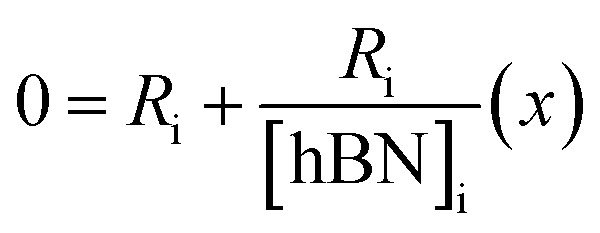
so,

6b [hBN]_i_ = −(*x*)

where *x* is the *x*-intercept of linear regression fit of the plotted data. Once [hBN]_i_ is obtained, [hBN]_f_ can be calculated for the “spiked” samples. The “spiked” samples, along with data points for pure h-BN and the FTIR data from Chee Huei *et al.*^9^ interpreted as “99% pure BNNTs”, can be used to establish a calibration curve of R/TO ratio *versus* [hBN]. In this way, [hBN]_i_ of any sample can be calculated from a calibration curve.

## Conflicts of interest

There are no conflicts to declare.

## Supplementary Material

NA-001-C8NA00251G-s001
