# Supplementary material for: Quantification of hexagonal boron nitride impurities in boron nitride nanotubes via FTIR spectroscopy
Source: Nanoscale Adv. 2019 Mar 12;1(5):1693–701. doi: 10.1039/c8na00251g (PMC9419701; doi:10.1039/c8na00251g)
Supplement: NA-001-C8NA00251G-s001 [file NA-001-C8NA00251G-s001.pdf]

Electronic Supplementary Material (ESI) for Nanoscale Advances.
This journal is © The Royal Society of Chemistry 2019

Quantification of Hexagonal Boron Nitride impurities in Boron Nitride Nanotubes via FT-IR Spectroscopy

Supplemental Information

Haley Harrison,[a] Jason T. Lamb,[b] Kyle S. Nowlin,[d] Andrew J. Guenthner,[c] Kamran B. Ghiassi,[c] Ajit D. Kelkar,[d] Jeffrey R. Alston[d]*SI Figures

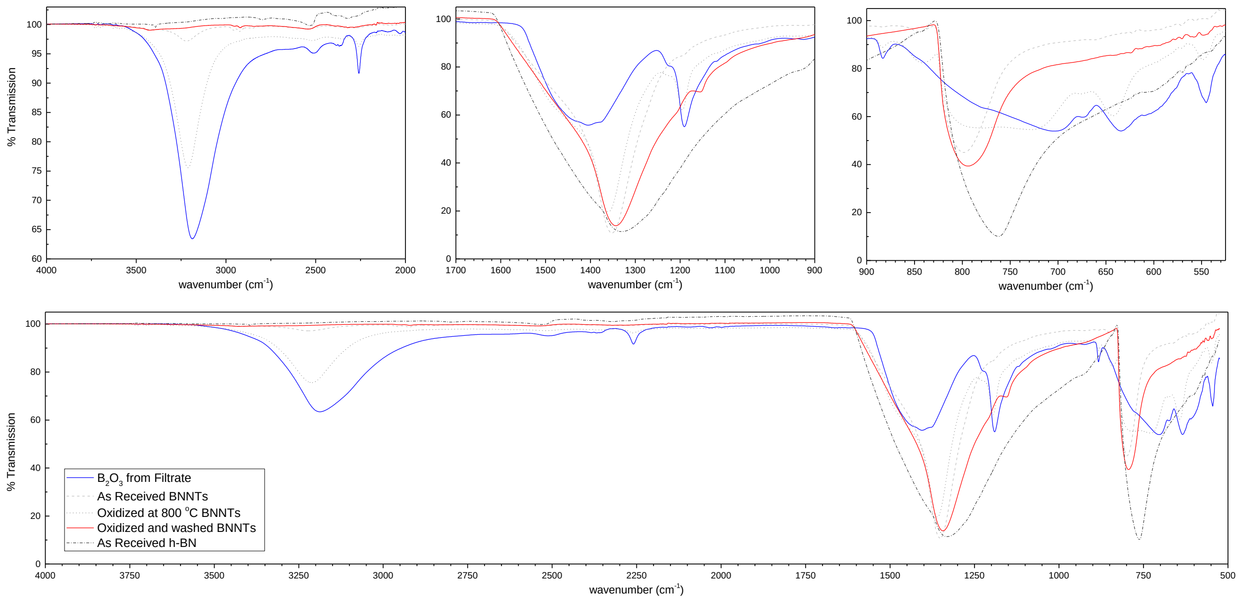

Figure SI 1. FT-IR transmission spectra of boron oxide overlaid on as received BNNTs and h-BN.

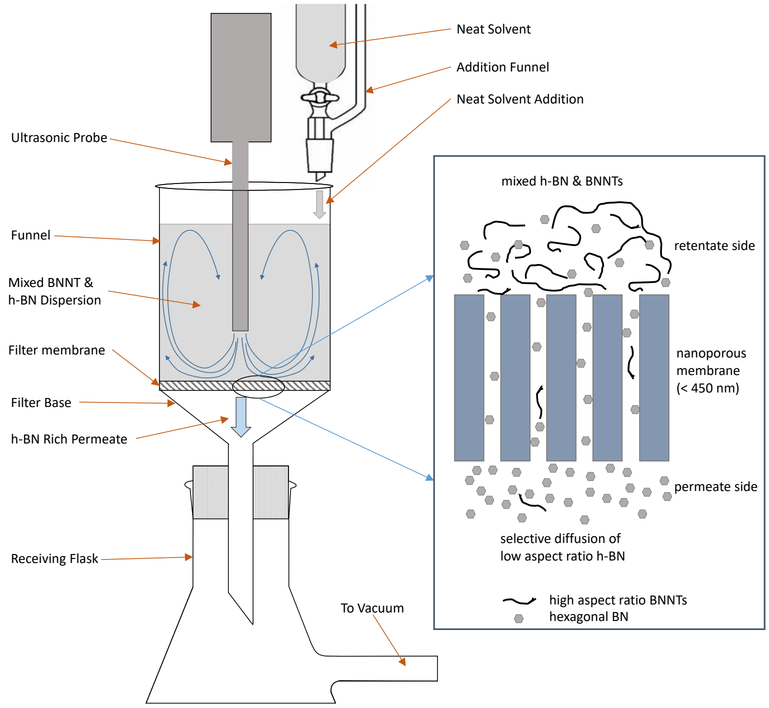

Figure SI 2. Schematic diagram of ultrasonically assisted diffusion separation of BNNTs and h-BN.

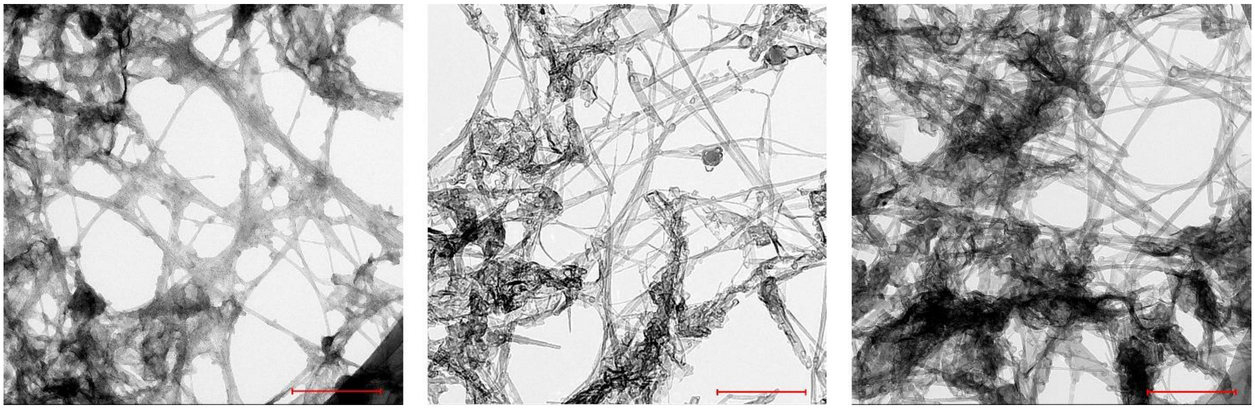

Figure SI 3. TEM micrographs of BNNT samples. From Left to Right, as-received BNNTs (AR-BNNTs), oxidized and washed BNNTs (OW-BNNTs), and Triton X-100 separated BNNTs (TX-BNNTs). The red scale bar for each image is 200 nm.

Useful Literature Images Provided for Analysis Perspective

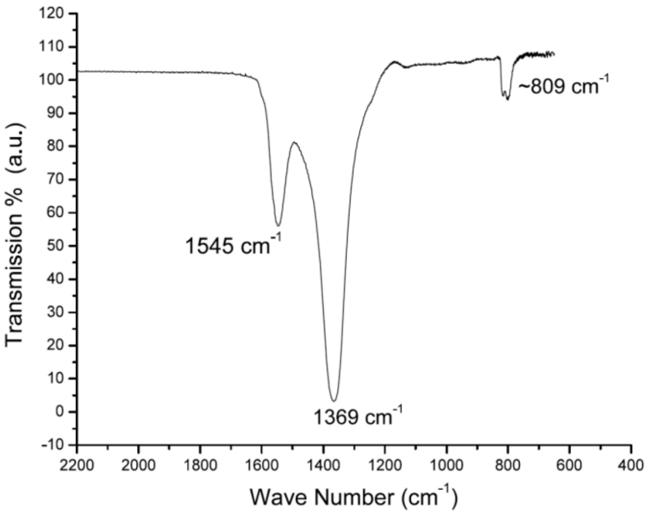

Figure SI 4. FT-IR transmission spectrum from highly pure boron nitride nanotubes (BNNTs). Reproduced from Chee Huei et al.[1] Vibrational modes along the tube axis (LO) of a BNNT correspond to ~1369 cm^{-1}, while circumferential or tangential vibration modes (T) tangent to the axis are at ~1545 cm^{-1}. Out-of-plane radial buckling modes are at ~809 cm^{-1}.

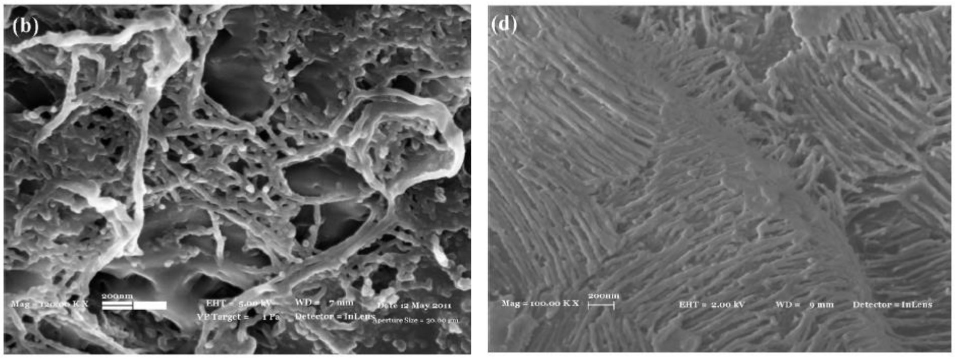

Figure SI 5. HRSEM image from Okan et al. Figure 9b sample BNNT-3 & Figure 9d sample BNNT-5. Representative SEM images of the BN structures formed during the synthesis described in the study.[4]

Boron Oxide and Boric Acid

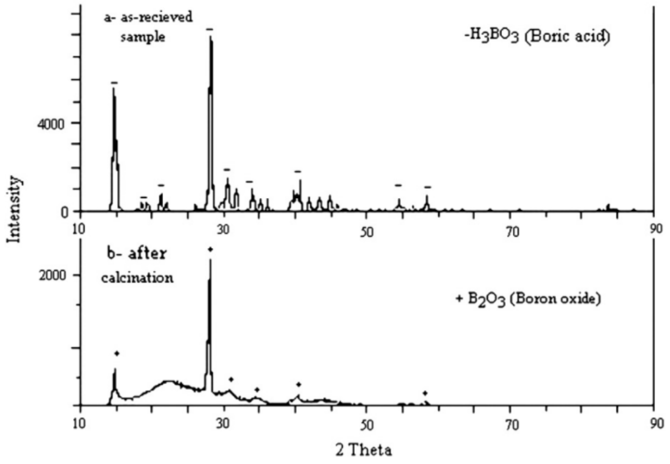

Figure SI 6. XRD patterns of a) boric acid and b) boron oxide powder after calcination of boric acid in $Ar_{(g)}$ at 400 °C.[2] A notable observation is the presence of a 2θ diffraction near 27°, which has been assigned to boron oxide (b) but overlaps similar peaks for boric acid (a), and peaks commonly assigned to (002) h-BN in many studies (Figures 6 and 7 of the main text).

Hexagonal Boron Nitride (h-BN)

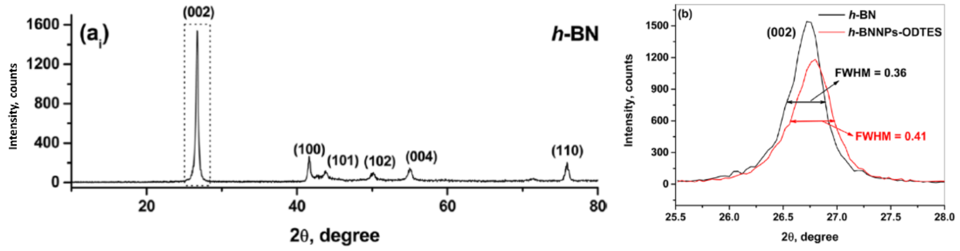

Figure SI 7. XRD Spectra of pure hexagonal boron nitride (h-BN), reproduced from Figure 3a_i and 3b in Kumari et al.[5] a_i) The diffraction peaks corresponding to the (002), (100), (101), (102), (004), and (110) planes are all clearly shown and is characteristic of highly ordered h-BN structure. b) The (002) peak is symmetric and narrow at a 2θ = 26.75° with a FWHM of 0.36°.

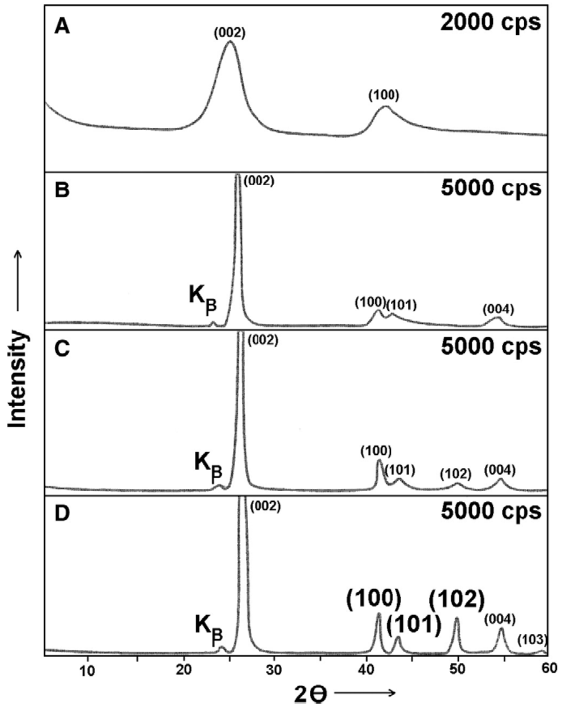

Figure SI 8. XRD spectra reproduced from Thomas et al.[6] and Figure 1 in Balint et al.[3] Resolution of the characteristic peaks of h-BN increase in order from A – D; showing the effects of three-dimensional ordering, from turbostratic boron nitride to complete three-dimensional order, respectively.

Boron Nitride Nanotube (BNNTs)

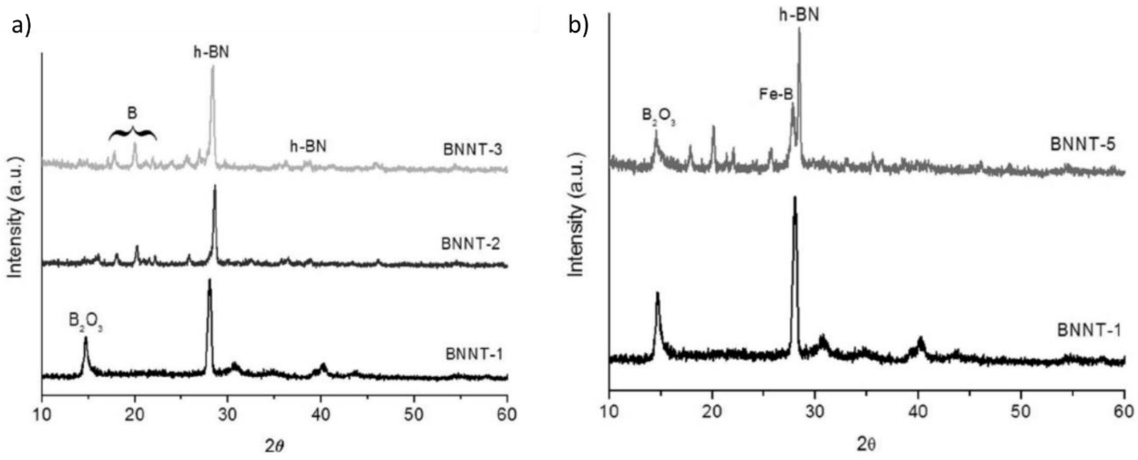

Figure SI 9. a) XRD patterns of BN nanostructures produced using pure boron powder. Diffraction peaks of (002) h-BN are observed at 2θ = 27.7°, 28°, and 28.4° for BNNT-1, BNNT-2, and BNNT-3, respectively. A diffraction peak of (100) h-BN is observed at 2θ ≈ 41° in BNNT-1. B_2O_3 is observed in BNNT-1 at 2θ ≈ 14°, while unreacted Boron in BNNT-1 and BNNT-2 is observed between 2θ = 15° – 25°. b) A diffraction peak of Fe-B, a high temperature (750 °C) side product appears at 2θ ≈ 27° for BNNT-5, compared to BNNT-1 produced at a lower temperature (600 °C).[4]

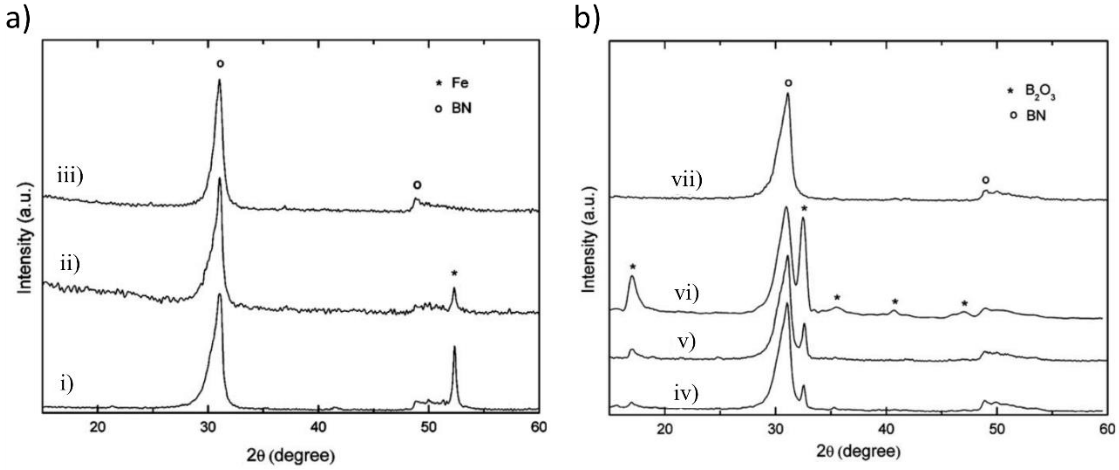

Figure SI 10. XRD patterns of BNNTs i-iii) as-synthesized, dispersed, and acid washed, respectively; iv-vi) oxidized at 750 °C, 800 °C, and 850 °C, respectively; vii) sample (v) washed with hot water.[7] Typical diffraction peak of (002) h-BN appears around 2θ ≈ 31° in all samples, and B_2O_3 is observed at 2θ ≈ 16° for oxidized samples.

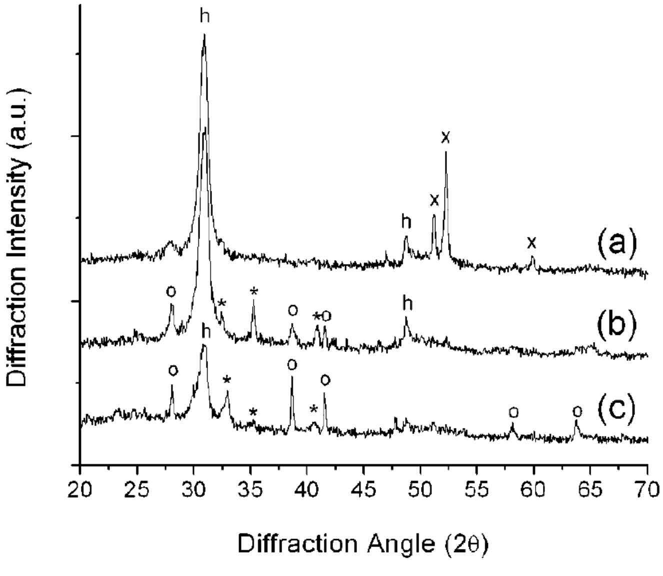

Figure SI 11. XRD spectra of BNNTs a) before oxidation, b) after oxidation at 700 °C (air), and c) further oxidized in air up to 900 °C. Peak designations are X: Fe; ○:Fe_2O_3 and *: B_2O_3, although not including 2θ angles below 20° in the spectra, to show the 2θ ≈ 15° formation of boron oxide is a crucial omission from this data.[8]

Figure SI

1. Chee Huei L, Jiesheng W, Vijaya KK, Jian YH, Yoke Khin Y. Effective growth of boron nitride nanotubes by thermal chemical vapor deposition. *Nanotechnology* 2008, **19**(45)**:** 455605.

2. Atasoy A. The aluminothermic reduction of boric acid. *International Journal of Refractory Metals and Hard Materials* 2010, **28**(5)**:** 616-622.

3. Balint MG, Petrescu MI. An attempt to identify the presence of polytype stacking faults in hBN powders by means of X-ray diffraction. *Diamond and Related Materials* 2009, **18**(9)**:** 1157-1162.

4. Saner Okan B, Kocabaş ZÖ, Nalbant Ergün A, Baysal M, Letofsky-Papst I, Yürüm Y. Effect of Reaction Temperature and Catalyst Type on the Formation of Boron Nitride Nanotubes by Chemical Vapor Deposition and Measurement of Their Hydrogen Storage Capacity. *Industrial & Engineering Chemistry Research* 2012, **51**(35)**:** 11341-11347.

5. Kumari S, Sharma OP, Gusain R, Mungse HP, Kukrety A, Kumar N*, et al.* Alkyl-Chain-Grafted Hexagonal Boron Nitride Nanoplatelets as Oil-Dispersible Additives for Friction and Wear Reduction. *ACS Applied Materials & Interfaces* 2015, **7**(6)**:** 3708-3716.

6. Thomas J, Weston NE, O'Connor TE. Turbostratic1 Boron Nitride, Thermal Transformation to Ordered-layer-lattice Boron Nitride. *Journal of the American Chemical Society* 1962, **84**(24)**:** 4619-4622.

7. Chen H, Chen Y, Yu J, Williams JS. Purification of boron nitride nanotubes. *Chemical Physics Letters* 2006, **425**(4–6)**:** 315-319.

8. Chen Y, Zou J, Campbell SJ, Caer GL. Boron nitride nanotubes: Pronounced resistance to oxidation. *Applied Physics Letters* 2004, **84**(13)**:** 2430-2432.
